# A Blockchain-Based Consent Platform for Active Assisted Living: Modeling Study and Conceptual Framework

**DOI:** 10.2196/20832

**Published:** 2020-12-04

**Authors:** Pedro Elkind Velmovitsky, Pedro Augusto Da Silva E Souza Miranda, Hélène Vaillancourt, Tania Donovska, Jennifer Teague, Plinio Pelegrini Morita

**Affiliations:** 1 School of Public Health and Health Systems University of Waterloo Waterloo, ON Canada; 2 CSA Group Toronto, ON Canada; 3 Research Institute for Aging University of Waterloo Waterloo, ON Canada; 4 Department of Systems Design Engineering University of Waterloo Waterloo, ON Canada; 5 eHealth Innovation Techna Institute University Health Network Toronto, ON Canada; 6 Institute of Health Policy, Management, and Evaluation Dalla Lana School of Public Health University of Toronto Toronto, ON Canada

**Keywords:** health care, blockchain, Internet of Things, aging, informed consent, mobile phone, computing methodologies, computer security

## Abstract

**Background:**

Recent advancements in active assisted living (AAL) technologies allow older adults to age well in place. However, sensing technologies increase the complexity of data collection points, making it difficult for users to consent to data collection. One possible solution for improving transparency in the consent management process is the use of blockchain, an immutable and timestamped ledger.

**Objective:**

This study aims to provide a conceptual framework based on technology aimed at mitigating trust issues in the consent management process.

**Methods:**

The consent management process was modeled using established methodologies to obtain a mapping of trust issues. This mapping was then used to develop a conceptual framework based on previous monitoring and surveillance architectures for connected devices.

**Results:**

In this paper, we present a model that maps trust issues in the informed consent process; a conceptual framework capable of providing all the necessary underlining technologies, components, and functionalities required to develop applications capable of managing the process of informed consent for AAL, powered by blockchain technology to ensure transparency; and a diagram showing an instantiation of the framework with entities comprising the participants in the blockchain network, suggesting possible technologies that can be used.

**Conclusions:**

Our conceptual framework provides all the components and technologies that are required to enhance the informed consent process. Blockchain technology can help overcome several privacy challenges and mitigate trust issues that are currently present in the consent management process of data collection involving AAL technologies.

## Introduction

### Background

Society is currently moving into an age of ubiquitous and smart technologies, including mobile and wearable products, portable sensors, and diverse internet of things (IoT) solutions [1]. Examples of such smart technologies are smartphones, smartwatches, wireless blood pressure cuffs, wireless scales, smart thermostats, and smart homes, among others [2-4]. These devices have had a substantial adoption rate; for example, in 2016, 76% of Canadians owned a smartphone [5]. These devices have become a standard and are pervasive even in developing countries; for example, in Brazil, 57% of the population used a smartphone [6], and in Argentina, a similar proportion of the population, that is, 52%, used a smartphone [7]. According to a recent survey of 11 developing countries across 4 global regions, a median of 53% of the population of these nations have access to a smartphone with internet [8]. The wearable market has also experienced rapid growth: the number of global smartwatch users increased from 5 million in 2014 to 75 million in 2017 [9]. The fitness wearable market alone, comprising devices such as fitness bands, smart clothes, and eyewear (eg, smart glasses), has approximately 4 million users in Canada with a revenue of Can $ 290 (US $220) million [10].

These technologies have embedded sensors that can continuously and effortlessly monitor the health of users [11] by collecting data on vital signs [12], environmental variables [13,14], and behavioral metrics such as movement in the house [1]. The collected data can be analyzed for new insights into the health of individuals and populations [15].

### Active Assisted Living

One of the fields that IoT technologies and connected devices can greatly improve and support is active assisted living (AAL) [16], defined as “all technology, devices, and wearables connected to the Internet, that enable the collection and exchange of data, and are used for health monitoring or to enhance the daily life of individuals” [17]. The major goal of AAL technology is to help people with activities of daily living, leading them to a better, safer, and more productive life while minimizing the risk of injury and avoidable death [1,17]. Therefore, AAL technologies are usually designed to support vulnerable populations and older adults [1,17].

AAL has become increasingly important because of a shift toward older populations in the global age distribution. It is expected that by 2050, all developing countries, in addition to Latin America, the Caribbean, and most of Asia, will have a median age of at least 40 years [18]. In Canada, 10 years from now, for example, 1 out of 4 people will be aged over 65 years [19].

Aging well and in place requires the older adult population to be as independent, secure, and healthy as possible [1,17]. However, older adults experience declining health as they age and are more likely to develop some sort of impairment, making remaining at home a difficult task [20]. Although assistive technologies have long been used to help older adults with daily activities [20], the benefits of innovative connected devices for monitoring wellness and supporting aging-in-place are being realized only recently. Their sensing capabilities allow for health support, real-time data collection, and detection or warning for emergencies. For example, 1 out of 4 seniors fall each year, resulting in the deaths of 27,000 seniors every year in the United States alone [21]. Another example of how these technologies can support older adults is a smart thermometer that cools the house if the temperature increases to dangerous levels, preventing seniors from experiencing a heatstroke [1].

Controlling access and consent to all these data is still a great challenge in the current technological landscape [18,22-26].

### Informed Consent

Informed consent is defined as an “individual’s autonomous authorization of medical intervention or participation in research” [18]. Valid informed consent in research and treatment is composed of the following minimum requirements [18,27]:

1. Competence: defined as the ability to perform a task
2. Understanding: defined as the full disclosure of information pertaining to the situation
3. Voluntariness: defined as participation in the event without any coercion and awareness of the possible outcomes.

Additional complexities in the consent management process may arise depending on the case; for example, older adults use AAL systems tend to interact with caregivers, cohabitants, and legal guardians. AAL technologies, such as smart thermostats, will collect data from every person in the environment where they are located. Therefore, the caregivers, cohabitants, and legal guardians must also give consent for data collection and use [18,23].

In addition, AAL technologies have significant benefits for people with cognitive impairments, including older adults and people with dementia [18]. A delicate ethical consideration arises on whether these populations can provide valid informed consent based on the principles outlined above [18]. One solution would be *rolling informed consent*, where the data collector repeatedly provides information on consent while assessing the mental capability of an individual [18,28]. Although rolling informed consent may work well in research environments, in practice, it may not be possible to achieve it in a real-world deployment of AAL systems because of the high complexity, rate, and volume of data collection from devices.

In cases where the individuals whose data are being collected cannot give valid informed consent, substitute decision makers (SDMs) may be necessary to make health decisions on their behalf [29]. SDMs may be family members, caregivers, legal guardians, or any person authorized to make health decisions for or with the individual.

### Privacy of AAL Data

Despite the benefits to technology users, health care providers, and the health care system, one major challenge that needs to be addressed is the privacy of patient-generated health data. Although IoT devices allow continuous and zero-effort monitoring of health data, they also increase the complexity of data collection points and make it harder to determine exactly what, why, and how data are being collected. This is especially troublesome when we consider the context of AAL technologies: older adults are a vulnerable population that traditionally do not have advanced technological knowledge [16,23,30,31].

Older populations using AAL technology are at a high risk of being subjected to security and privacy violations because of the mishandling of their data, in which mishandling is characterized by any use of data that are different from what was consented to by the user. Older adults (and technology users in general) must know what they are consenting to and how to manage their consent at any time. In addition, many technologies only allow a *binary* consent in which users consent to all data or no data being collected. Users do not have any choice of which health variables they can give or revoke consent to.

### Blockchain

Data ownership, security, anonymity, and privacy are complex topics, and, as exemplified above, the challenges of obtaining consent for increasingly advanced methods of data collection, use, and disclosure call for new solutions to imperfect consent procedures to protect the safety of individuals. One possible solution is using blockchain.

This technology can be seen as a distributed ledger formed of data structures known as blocks, equipped with cryptography techniques to enable trust among parties while being operated by a peer-to-peer network of computers [1,32]. Each computer forms an independent node on the network and maintains a copy of the ledger, which is regularly updated to ensure that it remains consistent with the other copies.

A blockchain can be private or public, depending on its intended application. A public blockchain is open to anyone who wishes to browse its contents or participate in the network [33]. Public blockchains are the most well-known blockchain applications because most cryptocurrencies take advantage of their features to enable trusted monetary transactions without the need of a trusted third party. In contrast, a permissioned or private blockchain allows only authorized users to browse its contents or participate in the network [34]. This type of blockchain solution is appropriate for sensitive or highly regulated information management environments, such as health care data.

A blockchain network receives transactions when two or more users want to transact information between them. Transactions from users are broadcasted to the network, validated, and grouped into a block by network nodes known as *miners* [35,36]. The transactions of a blockchain are data structures modeled to represent real-world processes and objects. For example, a transaction can be modeled to represent monetary transactions [37]; transfer of the ownership of a car [38]; the current state of a business object [39]; or, in our framework, the current state of informed consent of a patient.

Transactions sent to the ledger are secure and private without the necessity of a trusted third party because users of a blockchain do not use personally identifiable information as credentials when sending transactions to the ledger [1,32]. They use techniques such as cryptographic proof of ownership, in which every user has a private and public key pair for each transaction they submit to the ledger [40].

The sender signs the transaction with the private key, and a unique signature is generated and sent, along with the information of the transaction, to the public key of the receiver in the network. Every new blockchain transaction is broadcasted to all miners of the network who concurrently verify the transaction for proof of the private key ownership of the sender and verify whether the contents of the transaction are valid. For example, in the blockchain of Bitcoin [35], all transactions are checked for the private key ownership of the sender to ensure that the sender has enough funds (bitcoins) to send the transaction.

Miners utilize the public key of the sender to verify whether the signature of a transaction is valid. If the signature is not valid, it indicates that the original signature of the sender is wrong or tampered with, resulting in the network rejecting the transaction. The proof-of-ownership method prevents transactions from being corrupted or tampered before being added to the blockchain [34].

After transactions are validated, miners group them in a block; however, before adding it to the blockchain, they must complete a task known as mining [37]. Mining is a process in which miners compete to create a unique hash string for a new block.

A newly generated block hash contains in its composition the unique hash of the most recently added blockchain. The linkage of blocks’ hash creates a cryptographical heritage that enables blockchain to tamper-proof its information history. For example, if an attacker wants to change the transaction information contained in block number 50, and miners from the network are currently working on block number 100, the attacker must generate the unique hash of all blocks that come after block number 50 until they reach the end of the chain and generate the unique hash of block number 100 before all the other miners of the network finish working on it. For this type of attack to be successful, a significant amount of processing power from a single miner is required. More precisely, a single miner would have to produce more processing power than the entire network to achieve success. Hence, these types of attacks are improbable [41,42].

In typical cryptocurrency blockchain solutions, such as Bitcoin, miners compete to finish mining a block because the winner gets rewarded for completing the task. This process is known as proof-of-work [35], and one of its major disadvantages is the vast amount of electrical power needed to keep networks running. Newer blockchain solutions, such as Ethereum, take advantage of modern validation processes such as proof-of-authority. In this process, instead of wasting computational power to validate and hash a block, credentials or any other relevant fact about the miner is used to accept a new block as valid or not.

Proof-of-work and proof-of-authority are used by blockchain technologies to help the network achieve consensus. In all consensus methods, a consensus is achieved by the ledger when the majority of nodes in the network agree that the block is valid and add it to their local copies. The consensus process starts when the first miner in the network finishes mining a block. It first adds the new block to its copy of the ledger, then follows to broadcast the new block to its neighboring nodes. Each node that received this new block verifies the unique block hash for validity, and if it is valid, it adds the block to its copy of the ledger. The node then follows to broadcast the new block in the same manner as the miner. This process is repeated until part or all of the network agrees with the current block. Some blockchain solutions define that when more than 50% of the network agrees with a block, consensus is achieved. There is a plethora of blockchain solutions that offer different types of consensus mechanisms. As for our framework detailed in the next sections, we used hyperledger fabric (HF) [38] to take advantage of the pluggable consensus feature, which allows for future changes in the consensus model. This feature is essential to our framework because we cannot instantiate an application that is not capable of adapting to comply with future regulations. More details on the consensus mechanism of HF are presented in the *Results* section.

This paper explores the creation of a blockchain platform for consent management in health care, specifically in the context of AAL. We discuss a general methodology for identifying blockchain use cases developed by Gorenflo et al [43], and we apply this methodology to consent management for AAL technologies. With the identification of consent management as a prime use case for blockchain, we expand upon previous work done by Bublitz et al [1], deriving from their general software architecture for surveillance activities, a conceptual framework for blockchain in AAL consent management. To develop this proposed conceptual framework, we researched which blockchain technology was the best fit. In the following sections, we discuss the methodology for identifying trust issues and software architecture, which served as a basis for our work. Next, we present our framework and explain how it differs from related work.

## Methods

### Mapping of Trust Issues

Gorenflo et al [43] defined a general methodology for identifying use cases of blockchain based on the identification of trust relations. This robust approach involves the following sequential steps relevant to this paper:

1. Identify the parties and trust relations between them. If a relationship does not have the required level of trust necessary to achieve the goal of the relationship, it should be marked as a trust issue.
2. Design a minimal blockchain system that resolves the trust issues.
3. Migrate the rest of the existing system to the new blockchain system if such a system exists.

**Figure 1:**
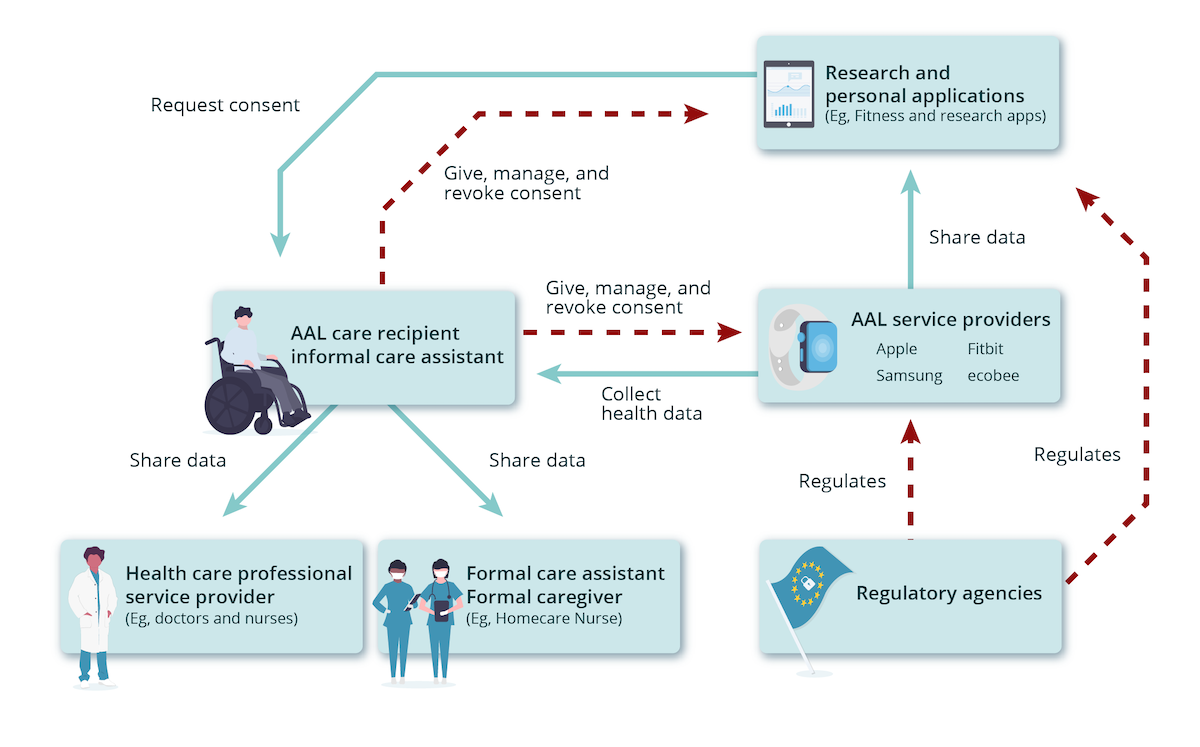
Consent management process and trust issues in active assisted living. AAL: active assisted living.

In this study, we followed this methodology to model the consent management process for data collection in the AAL technology space, in consultation with AAL experts, resulting in the diagram presented in Figure 1.

### Framework Development

**Figure 2:**
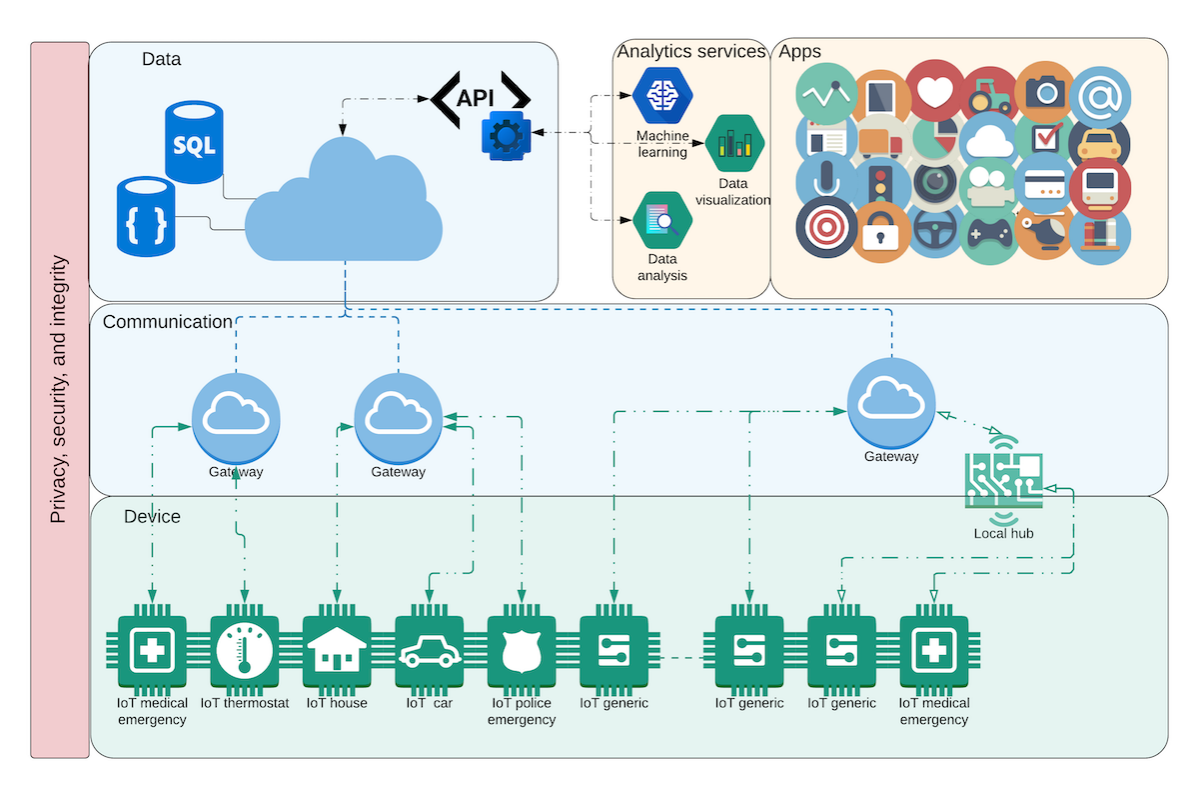
General architecture for the pan-Canadian surveillance system. API: application programming interface; IoT: internet of things; SQL: structured query language.

Once the trust issues were identified, we began work on a conceptual framework that focused on blockchain to mitigate these issues. This framework is based on a general framework architecture for monitoring and surveillance activities created by Bublitz et al [1] to address most of the requirements for the creation and use of IoT systems. The main layers of the architecture, as exemplified in Figure 2 [1], are device, network, data, applications and privacy, security, and integrity.

## Results

### Trust Diagram for Consent Management

The trust diagram in Figure 1 follows the nomenclature from the International Electrotechnical Commission or Systems Committees Active Assisted Living [1,17,44]. The identified trust issues are highlighted in red on the arrows included in the trust diagram.

An AAL Care Recipient (or an Informal Care Assistant, a nonprofessional caregiver) uses AAL technology. These users need to manage their consent to data collection and use by the manufacturers or owners of the technology, the AAL Service Providers, and any third-party application that collects the data gathered from the technology, represented as Research or Personal Applications. Trust issues arise between the AAL Care Recipient and entities consisted of Research or Personal Applications and AAL Service Providers because these entities may use the collected data in different ways than what was consented to. Another trust issue between these entities and regulatory acts, such as the Personal Information Protection and Electronic Documents Act (PIPEDA; in Canada), Health Insurance Portability and Accountability Act (HIPAA; in the United States), and General Data Protection Regulation (GDPR; in the European Union), which regulate the collection of personal data, is that the entities may be illegally using the data regarding the current legislation, thus violating the rights of the users.

The diagram clearly demonstrates relations without the necessary level of trust in the consent management process. Blockchain technology can provide an immutable and timestamped log of consent, making the process more transparent for everyone involved. This is extremely important as consent management, traditionally, is subject to many shortcomings and misconducts [45], and not following correct consent procedures can have tragic consequences. For example, in 2016, the trial testing of the drug BIA 10-2474 in France caused the death of a participant and hospitalization of 5 others, an event classified as that of “exceptional gravity, unprecedented in our country” by the Minister of Solidarity and Health [46]. Preliminary investigations showed that after major neurological effects were discovered in 1 patient, the researchers did not obtain reconsent from other participants, allowing them to continue in the trial despite clear dangers to their safety [46]. If the researchers used a platform such as the one proposed here, all their interaction with the participants regarding consent management would be recorded with a timestamp, making it extremely difficult for the researchers to not obtain reconsent as this misconduct would be easily auditable.

With a blockchain platform for consent management, users are able to monitor and manage their consent in real time and with granular variable control, for example, by giving informed consent for certain types of data to be collected but not others or revoking their consent at any time. This is in alignment with privacy regulations, such as the data protection by design of GDPR and right to data deletion by users at any time [47]. This information will be immutably stored in the blockchain and may be accessed by all entities with permission to do so. Users will also be able to ensure that the forms were approved and cryptographically signed by the review ethics boards, ensuring that the researchers are not withholding any information.

Data collectors will also benefit as measures taken to ensure ethical and legal requirements throughout the data collection process will be clearly documented and auditable.

A feature of blockchain called smart contracts [1] can also be leveraged to improve the consent management process. Smart contracts can be seen as codified contract agreements, and because blockchain is an immutable ledger, terms of a contract written into software and embedded in the blockchain will always guarantee the fulfillment of these terms [1]. Smart contracts can be used, for example, to bind SDMs to an individual or to check whether the consent process is compliant with different privacy regulations across territories. Ultimately, with blockchain technology, it will be easier to obtain, track, and update informed consent.

### The Blockchain: HF

The HF [48] platform was selected for use as the underlying blockchain technology as it provides the tools to achieve the goals of our proposed conceptual framework (Textbox 1). HF is an open source, permissioned, distributed ledger technology (DLT) platform [38,49] developed to be used in the contexts of enterprise. All enterprise interactions are performed in a private network environment called hyperledger fabric network (HFN). An HFN provides blockchain services so that client applications and network administrators can interact with the HFN, proposing new transactions and querying the blockchain. In an HFN context, a consortium [50] comprises 2 or more organizations on the HFN that need to transact business with each other.

In HF, an organization [51] is a logical driven group of members that can represent big or small corporations. Each HFN organization is composed of members called peers [52]. Peers are responsible for hosting ledgers, smart contracts, handling changes to the ledger, communications between different organizations (channels), and managing external requests from applications. An endorsing peer is responsible for executing smart contracts over a transaction proposal from a client to verify its validity before it can be added to the ledger. A committing peer is a member who keeps a copy of the ledger without any smart contracts, meaning that this is done to keep multiple copies of the ledger to avoid single-point failures. Ordering peers and organizations [53] are responsible for keeping the communication sound between the participants of a channel; however, those components are beyond the scope of this paper.

Organizations can communicate with each other on an HFN by creating and using communication channels. Communication channels are private communication environments for all or a subset of the organization’s members of a consortium. New organizations are enrolled in the HFN through a trusted membership service provider (MSP) [54], which is responsible for issuing and validating certificates and user authentication.

The conduct of business transactions between organizations of an HFN consortium is stored in a ledger. A ledger stores both the current state and the history of states from a business object. To keep a business object, the current state HF uses a database called the world state [55], which is explained later in this paper. To store the history of the transactions of a business object, HF utilizes a blockchain data structure [38]. Together, the blockchain structure and world state are what allow the ledger to hold an immutable history of states of business objects (blockchain) and provide fast access to the most recent state of a single business object.

Participants of the HFN achieve consensus by following a transaction flow that takes into account the endorsement policy assigned to smart contracts [56]. This means that transactions can only be accepted into the ledger if all, or a subset of, the endorsing peers of a consortium approve the transaction. Smart contracts in HFN are a software representation of a contract that governs the processes that alter the state of a business object. In HF, smart contracts are packaged into a structure called chaincode, which consists of multiple smart contracts. Each chaincode is deployed into an HFN channel with an endorsement policy assigned to it. The endorsement represents which organizations must sign a transaction so that it is accepted and added to the ledger.

This transaction flow from HF relies on identity validation for members of the network. All transactions are first sent to the network as a proposal that needs to be endorsed by all HFN organizations included in the policy of the smart contract. The proposal is signed using the cryptographic credentials of the user that generate a unique signature.

Endorsement organizations from HFN verify transactions for several factors: (1) a well-formed transaction proposal, (2) whether the proposal is not repeated, (3) checks with the MSP [54] to verify whether the proposal signature is correct, and (4) then the MSP verifies whether the identity of the proposal submitter has the authorization to perform operations in that channel.

Input parameters are fed to the chaincode that is going to be executed in the current world state. The result is returned after chaincode execution alongside the peer signature to the client as a proposal response. Finally, the client inspects the response from peers, and if all the necessary signatures are valid, the transaction is created.

The client application broadcasts transactions to the ordering service. The service, in turn, creates orderly blocks of transactions and sends them to the channel’s peers. Each peer verifies the block transactions and signatures before adding the block to the ledger. HF is not the only appropriate platform that can provide the necessary infrastructure to instantiate our conceptual framework. For example, Ethereum [57] is a blockchain solution that provides full support for distributed applications over a blockchain network. Even if the main network of Ethereum is public, which is not ideal for the health care domain, it can be instantiated into a private network environment, thus becoming compliant with the privacy and access control requirements of the present conceptual framework. We refer to Modum [58] as an example of an implementation of a supply chain management system applied to Ethereum. Modum enables pharmaceutical companies to monitor the temperature and humidity of medical products by using smart contracts to verify sensor data during their life cycle.

Other honorable mentions of blockchain platforms that are appropriate to this framework instantiation are the IBM blockchain platform [59], the Multichain Private blockchain [60], the Hydra chain [61], and the BigchainDB [62]. It is also worth mentioning that using these technologies will require some changes in the HFN [63] component of the framework.

Textbox 1 contains the minimum requirements of the solution mapped over the attributes of HF, providing a checklist for other blockchain platforms to instantiate the framework.

**Active assisted living conceptual framework requirements and features of hyperledger fabric that support them.**

**Privacy of patients’ information**

- Hyperledger fabric (HF) uses cryptographic material to control access to the ledger. Applications from authorized organizations keep information from patients in their infrastructure. HF only stores anonymized consent information from unique keys created for each patient.

**Private communications between stakeholders of the informed consent process**

- HF maps stakeholders from a network into organizations. Each organization can be a part of a channel composed of some other organizations that constitute the consortium. Communications inside a channel are private to their members.

**Scalable and fast querying of the ledger**

- HF stores the most recent state of a business object in a state database called world state. Every time an informed consent state changes or a new informed consent is created, a new transaction is inserted into the ledger. However, the world state only updates the business object if it already exists, otherwise it is created. These characteristics also make HF more scalable as querying the complete history of the ledger is not required for determining the most recent informed consent status.

**No cryptocurrency**

- HF has no cryptocurrency, which complies with our solution’s intention to provide the simplest solution possible to our end users, people using active assisted living.

**Security of information**

- As HF is a private and permissioned blockchain platform, the security of information is much less susceptible to a privacy breach.

**Immutability of ledger history**

- HF provides an immutable ledger natively.

**Accountability of informed consent transactions**

- With HF, smart contracts can be created enforcing standards. These smart contracts can be configured to explicitly require the endorsement of an organization that represents an auditing authority.

**Software development kit (SDK) for development of end user applications capable of interacting with the ledger**

- For our conceptual framework to be instantiated, the creation of web applications capable of interacting with the blockchain network to create and retrieve consent transactions is necessary. HF provides such SDKs in NodeJS, Java, and Go languages.

**Smart contracts capabilities**

- Smart contracts are needed to ensure the enforcement of standards and endorsements that must be respected so that a new informed consent transaction is accepted and stored in the ledger.

**Custom network policies**

- Such policies are needed to control network interactions between organizations. The informed consent process has its own unique policing needs, such as endorsement policies, hence the need for customization.

**Extensibility of the network**

- For our solution’s relevancy, the capability of adding new organizations, policies, channels, and smart contracts without having to start a new network is imperative. HF provides the plug-and-play capability for adding new entities into an existing network.

### Conceptual Framework for a Blockchain Consent Platform in AAL

**Figure 3:**
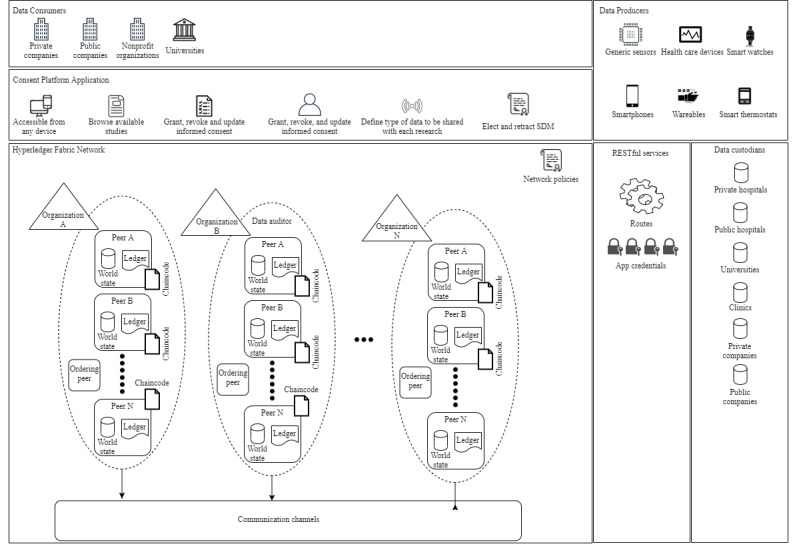
Consent management platform conceptual framework. SDM: substitute decision maker.

As previously mentioned, the main objective of the conceptual framework is to allow a plethora of smart *devices* data to be used by different health care stakeholders while providing trust, security, and privacy. Figure 3 illustrates the conceptual framework components, one for each domain of services and health care data. All stakeholders are either mapped into an HF organization or are end users that interact with the framework through the web application of the consent management platform. Each square in Figure 3 represents a different framework component, and each component maps services, applications, participants, and infrastructures that need to interact with the platform to complete the instantiation of the framework.

For example, the data consumer component interacts with the consent management platform component to create requests for informed consent from AAL patients. The data producer component must interact with the consent management platform component to grant informed consent to data consumers and to register the consent into the ledger. The consent management platform component interacts with the RESTful (representational state transfer) services component to send transactions to the ledger. The RESTful services component acts as the middleware between the platform front-end applications and the HFN component.

Finally, the data custodian component interacts with the consent management platform to ensure that a data consumer has the proper informed consent registered in the blockchain before sharing patient data.

The *Security* *Model* section discusses and explains the platform security aspects in details (Figure 3), along with what features from HF were utilized to support the goals of the framework.

### Security Model

As seen in Figure 3, the conceptual framework is built on top of an HFN; hence, all communications between organizations and their clients are secure by state-of-the-art authentication methods such as x509 certificates and asymmetric key pairs [38]. We derived our security model from the architecture of HF because it offers cryptographic certificates and keys necessary to access the ledger.

The consent management platform application utilizes HF ordering service [53] to access the HFN. HF applications are bound to an organization, and each time a new transaction is sent from an application, the network verifies the credentials of the organization and users before accepting transactions. If the credentials of the HF application are valid, then the transaction can be submitted to the ledger.

All transactions sent to the ledger are previously checked by cryptographic access control guards, which ensures that only authorized users can access the HFN. Chaincode is used to ensure that all transactions are signed and verified by the necessary endorsing organizations. For each organization endorsement, digital signatures are used to provide data authenticity before adding the transaction into the ledger. If an endorser organization rejects the authenticity of an application’s user, then the transaction will not be inserted into the ledger.

Cryptographic authentication mechanisms are simplified by a feature from HF called MSP [54]. An MSP abstracts complexity involved in issuing, validating, and certificating users’ and organizations’ identities. An MSP is configured to have unique identities and rules that govern these identities. The HFN utilizes the identities of users to control access to the ledger. For example, a user with a *client* type of identity can only transact on the network, but an identity of type *peer* is allowed to endorse or commit transactions to the ledger.

### Data Consumer Component

The data consumer component represents stakeholders that are interested in consuming data for a variety of different reasons, such as remote patient monitoring (RPM) [64-67]. To access health care data, obtaining informed consent from participants is essential to protect all participants, and ensuring safe and ethical procedures are in place is also essential [18,22,24,45]. The framework provides such interactions through data consumers interacting with the HFN component. It is also essential to explain that data collected for a data consumer can be real time or historical. This means that levels of security and awareness requirements from participants can differ, thus forcing the utilization of stricter network policies and smart contracts based on the characteristics of the data.

### Data Producer Component

The data producer component is constituted by stakeholders that provide health care data in the realm of the conceptual framework [37,68]. Collected data can be from passive or active monitoring [69]. The former relates to data that are continuously or periodically sent to the cloud (eg, smart thermostats), and the latter is the type of data that requires actions to be taken on the part of data producers (eg, clinical exam) [69]. Any request for active data use from a data consumer must be consented to by a data producer for a data consumer to gain access to it. Consent is given when a data producer interacts with the consent management platform and explicitly gives consent to the data consumer. The consent management platform is responsible for creating the transaction and sending it to the HFN. If the transaction is valid and endorsed by all the appropriate peers, then the transaction is stored into the ledger and the world state is updated.

### Data Custodians Component

The data custodian component contains stakeholders that are responsible for stored health care data [70,71]. Aside from the PIPEDA and HIPAA considerations with regard to security and privacy standards that all data custodians must adhere to, they must only provide access to data by another stakeholder from the informed consent process if the informed consent exists and if it has not expired. It is worth mentioning that a data consumer may also be a data custodian in some cases, but informed consent must be obtained in the same manner as previously noted. If a stakeholder is a data custodian, in the holistic view of the framework, this does not mean they are entitled to use health care data freely [69]. Data custodians are represented as HFN organizations in our framework, so that they can be part of the channels responsible for storing consent information. Therefore, by querying the world state, they can verify whether the consent given to a data consumer is valid.

### HFN Component

The HFN component represents the mapping of all the stakeholders of the conceptual framework into HFN organizations. By creating this mapping, it is possible to create consortiums capable of having private communication channels with their ledgers and smart contracts. These channels are used by the organizations to query, create, and update informed consent state transactions that are stored within the ledger. New organizations can be added to the HFN at any point; thus, the organizations in the framework architecture are generalized. The ledger contains historical data from all the transactions between the organizations, which allows for data auditing from any of the network participants, consequently ensuring that trust evolves as a result of design.

Another feature that comes by design, thanks to the use of HF, is the world state. The world state possesses the current values of a business object (in our case, informed consent data). This is necessary for our conceptual framework, as it would be expensive and time-consuming to go through all transactions stored in the ledger to find the most recent state of a consent document. In HF, the world state is a NoSQL (No Structured Query Language) database that stores key-value pairs as identifiers of each state. The key-value pairs can be, for example, the pair patientID-researchID. Every time a new transaction from this key is stored into the ledger, the key-value or pair state stored in the CouchDB will also be updated.

Finally, as per the current version of HF, we utilize the consensus mechanism from the Raft protocol. The Raft protocol is based on a leader and follower model in which a node is elected as leader and the rest as followers. The Raft protocol is also a crash fault tolerance service that complies with our requirements of ensuring access to patients’ consent information at all times [72].

### Data Auditor Component

A data auditor can be any organization that represents regulatory authorities such as the PIPEDA and HIPAA. Having data auditors present on the network is important because they ensure that law and regulations are enforced in the HFN by using smart contracts and endorsement policies. By being a participant of the HFN and possessing the endorsement of peers included in the proper channels of interest, for example, channels that store information of the data auditor’s jurisdiction, a data auditor can audit the information of multiple ledgers if needed. This HF capability allows the process of informed consent management to be auditable by different authorities, granting the conceptual framework the capability to adapt to new rules and policies that new data auditor added to the HFN might bring to the consortium.

Another possibility is that a data auditor, through the creation of policies, can force transactions to satisfy a set of requirements through smart contracts and network policies. Smart contracts enable the enforcement of standards for data privacy and security that prevent breaches of privacy and misuse of information. Through the proactiveness of a data auditor, it is possible to enhance the informed consent process as it can continuously verify the validity of consent. A data auditor can act upon expired or invalid consent information by being a member or founder of the channels in which the organizations under its jurisdiction are present.

### Consent Management Platform Component

The consent management platform is a web-based platform that groups all the functionalities necessary for the processing of granting, revoking, and managing consent. First, to prevent a lack of access from stakeholders because of technology limitations, the framework proposes the use of responsive web applications that can be accessed by any device. The platform uses HF’s NodeJS software development kit (SDK) [73] to interact with the HFN from the front end. Different endpoints can be used to control actions allowed only by certain types of users and organizations, for example, a patient (data producer) can elect an SDM to make decisions for him but cannot create requests for consent like a data consumer.

Patients can log into the platform to browse available data consumer requests and choose which to enroll in. For example, if an AAL patient wants to be monitored by an RPM center, the RPM center, as a data consumer, logs into the platform and creates the request for consent to be accepted by its patients. The AAL patient can grant consent to the RPM center through the platform’s web application. In doing so, a transaction is created and proposed to the HFN and, if accepted, is stored in the ledger. This ensures that all the stakeholders from the transaction now possess a copy from the current state of consent, and the data consumer can start collecting the patient data from data custodians.

The consent management platform also provides web interfaces that allow patients to revoke consent and update consent. Another feature offered is that data producers can choose to share specific types of data for each request for consent. For example, an RPM center may request 5 types of data to be shared by a patient. However, the patient may choose only to share 2 types if they desire; although this makes the expected data received by the data consumer less predictive, it empowers data producers at the same time. This process ensures accountability, security, and transparency for the informed consent process, helping to improve traditional methods of obtaining consent.

The consent management platform can also be used to enhance the process of electing SDMs for patients. A patient can request that a user becomes their SDM. If the appointed SDM accepts the request, the information about the SDM elected is stored into the ledger and becomes available to stakeholders. Just like an informed consent state stored into the ledger, the SDM state can also be revoked or updated based on the circumstances. Such features can help to empower people under AAL conditions to quickly elect trustworthy SDMs without having to go through current bureaucracies that govern the substitute decision-making process [74].

Finally, the representational state transfer services layer is responsible for managing appropriate access to the consent management platform and for keeping meta-information about the users’ identities in the ledger. If any other functionalities are needed, the conceptual framework can be extended to encompass new functionalities and components.

### Chaincode

The utilization of chaincode (smart contracts) by the framework ensures that only registered applications can send a transaction to the ledger and that the network’s required organizations endorse only valid transactions. A set of endorsement policies is defined for each new chaincode deployed into the network. As we are proposing a conceptual framework, at this stage of our research, we still do not have abstract contracts that can be extended by other developers. However, in the *Framework Instantiation* section, we explain the development of our prototype’s chaincode.

Our first implementation of the chaincode will serve as a subject to further enhancements for the framework. In future work, we will study all contracts and their commonalities to create abstract contracts that can be instantiated by developers who want to adapt the framework for other domains.

### Framework Instantiation

**Figure 4:**
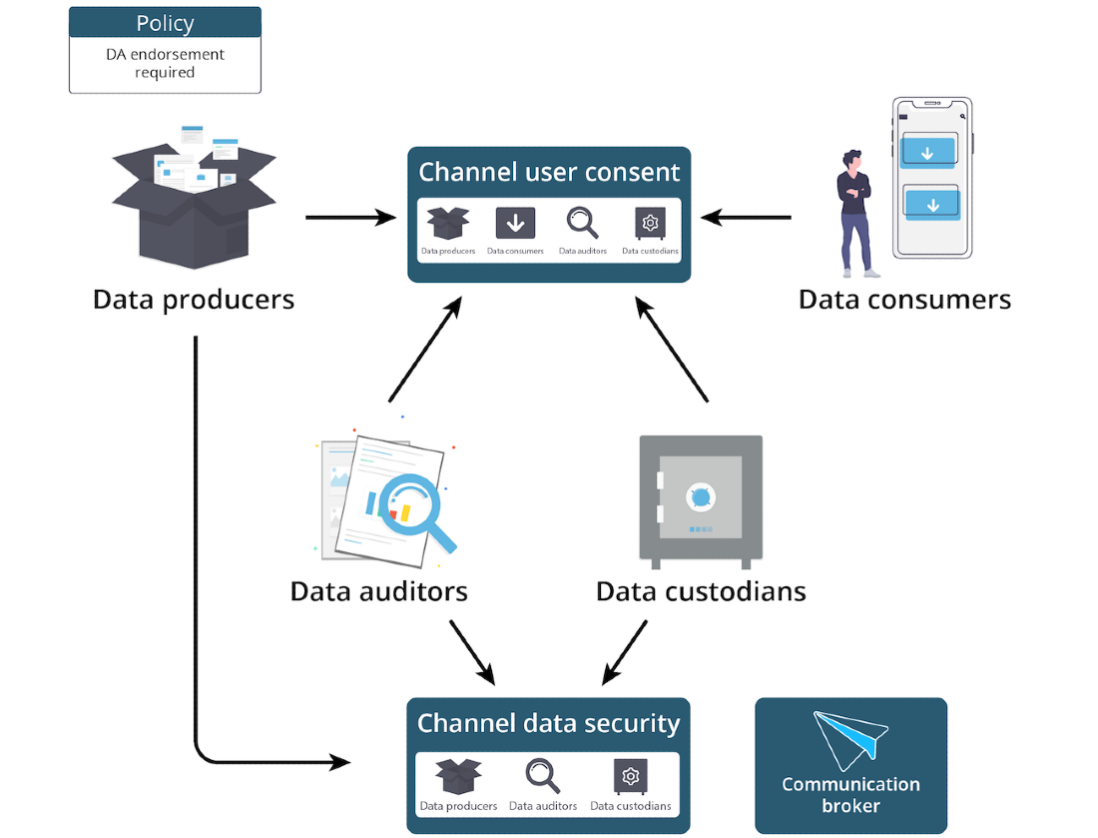
Organizations instantiated in the framework. DA: data auditors.

To exemplify the instantiation of the framework using HF, Figure 4 shows an abstraction of the developed HFN. The consortium is composed of 4 organizations that represent minimum requirements to allow all stakeholders in the system to perform the required tasks.

In our network, the 4 organizations that compose the consortium are data producer, data auditor, data consumer, and one data custodian. The members of the channel, channel user consent, have access to the ledger containing informed consent information. Before any new consent is given and stored into the blockchain, the data auditor member of the channel must agree and endorse the transaction containing the informed consent (as stated by the network policy in Figure 4). The data custodian, as a member of this channel, knows that the data auditor agreed to that consent if the transaction is valid and stored into the ledger. With the transaction validated, the data custodian is authorized to give access to its health care data to the data consumer as long as the data custodian respects the restrictions defined by a patient or SDM.

The data auditor organization is also a member of a different channel of communication called channel data security. This channel will hold the members of the network responsible for ensuring that the data custodian has the proper consent from users to store their information on their servers. Finally, the communication broker represents an organization responsible for routing the communication between the end user and the communication channels.

We used NodeJS SDK of HF to implement the chaincode responsible for verifying, adding, and querying informed consent of the patients and SDM states for members of the channel *User Consent*. The chaincode of the channel is defined by 2 contracts: contract one (C1) and contract two (C2). C1 is used to manage the informed consent state and to manage substitute decision maker states. C1 ensures that all fields of the informed consent transaction are present and valid. C1 checks for patient and research identification fields; the number of sensors; and, if for each type of sensor shared, the periods of consent are valid. C2 is responsible for managing SDM states from members of the *User Consent* channel. C2 checks for SDM and patient identification, start and end dates of the SDM validity, and the current status of the SDM-patient relationship. A code snippet implementation using NodeJS SDK of HF for C2 is presented below (Textbox 2).

If all requirements are successful, then the organizations, after executing the chaincode, endorse the transaction and return the proposal result to the sender. After finishing the proposal process, the new informed consent status is added to the ledger.

**Code snippet from the chaincode to add a new substitute decision maker.**

1. /**
2. @function addSDMState
3. @param {*} ctx
4. @param {*} patientID
5. @param {*} SDMID
6. @param {*} rlStatus
7. @param {*} startDate
8. @param {*} endDate
*/
10. async addSDMState(ctx, patientID, SDMID, rlStatus, startDate, endDate) {
11. try {
12. // Instantiate a new SDM state to be added to the ledger
13. let substituteDecisionMakerState = SubstituteDecisionMakerState.createInstance(patientID, SDMID, rlStatus, startDate, endDate);
14. if (substituteDecisionMakerState) {
15. // add to the ledger
16. const response = await ctx.substituteDecisionMakerStateList.addSDM(substituteDecisionMakerState);
17. // return ledger response
18. return response;
19. } else {
20. console.log(“Error at addSDMState”);
21. return null;
22. }
23. }catch (error) {
  console.log(“Error at addSDMState”);
24. }
25. }

## Discussion

### Enhancing Consent Management

Ensuring proper informed consent is a major concern for data collection and use [22,45]. Our proposed solution facilitates health data sharing by different stakeholders while increasing transparency and trust. The biggest advantage of our solution is that it was developed based on a systematic process of identifying and mitigating the trust issues in the consent management process [43]. The framework minimizes all trust issues indicated in Figure 1 by providing a virtual space to manage consent, powered by blockchain to provide an immutable and timestamped log of user consent for data owners and collectors. Consequently, AAL care recipients and informal care assistants will have a much better understanding and control over what data they are sharing, with whom, for what purpose, in what manner, and for what time period. Furthermore, it will be easier for regulatory agencies to audit if AAL service providers or research or personal applications are using data for purposes other than what was originally consented to, as they will be able to access the log of consent of the blockchain.

The US Food and Drug Administration (FDA) reported in an overview of clinical trial inspections from 1977 to 2009 that 28% of trials had deficiencies related to inadequate consent forms [45]. In 2012, the FDA cited the main deficiencies related to consent, among others: the failure to obtain informed consent; use of expired, incomplete, or nonvalidated forms; failure to provide copies of the forms to study subjects; missing documents; and changes made to documents by hand and without the approval of ethics review boards [22]. Our platform will provide a secure and immutable virtual space where all stakeholders—data owners, consumers, and regulators—will have complete transparency and surety of the entire process, making the process safer for all involved and ensuring that cases such as the trial of BIA 10-2474 will not happen again.

In short, the proposed conceptual framework tries to generalize the process of consent management for all stakeholders in the health care domain, allowing a clearer understanding of possible interactions and functionalities of the consent management process and, ultimately, providing more transparency. As presented by Novitsky et al [18], this was an important characteristic to help address inefficiencies in the process of consent management, especially for vulnerable populations.

The framework also aims to take full advantage of the features of HF to allow for robust and complex control over the process of obtaining informed consent. Data auditors are an example of such goals, in which they can remove bad actor organizations from the network if they do not comply with the rules.

### Related Work

Several companies explore consent management and blockchain. However, they differ significantly from our solution.

Hu-manity.co, for example, developed a mobile app with IBM blockchain to help individuals manage consent for the use of their personal and health information. This app gives users a title for their digital data, declaring them as the user’s property [75]. Unlike our proposed platform, this solution does not store the user consent information on the blockchain.

Another company, Bitfury, is producing a blockchain-based consent management system for research and medical data. This solution is closer to what we are envisioning as a blockchain platform [76,77]. The same is true for solutions in academia, such as the work of Benchoufi and Ravaud [26] that use blockchain to provide a timestamped log of consent for clinical trials. We differentiate from these works not only by focusing on IoT and AAL, and outside clinical and research contexts, but also by providing a granular variable control for users, allowing them to manage consent for different data types and periods (eg, user 1 gives consent for the temperature to be collected but not their movement, between June and July).

An interesting related solution is MedRec, developed by researchers at the Massachusetts Institute of Technology. Unlike previous works, this solution was not created to improve the consent management process but to minimize interoperability issues between electronic health records (EHRs) while providing a transparent view of a patient’s full medical history. MedRec uses smart contracts to encode metadata with references to medical data from multiple medical data sources, which includes information regarding ownership of the data. Patients permit data to be accessed and shared [25]. In MedRec, consent management is not an explicit concern, but it appears as a form of access control for medical data. Although our solution considers access to data, as described in the sections below, we focus on consent management and improvement of trust relations in this process. In addition, MedRec deals with medical data stored in clinical systems and already collected, whereas our platform deals with real-time, patient-generated health data from sensors and AAL technologies deployed in the real world. Although our solution is distinct from solutions that deal with EHR interoperability, having proper informed consent is always a concern when dealing with personal data. Our proposed solution is a possible facilitator such that future health data sharing solutions do not need to develop tools to manage informed consent.

### Limitations

The limitations of this work include the lack of implementation and real-world deployment of our proposed conceptual framework. Therefore, future work will focus on the development of a blockchain-enabled platform for consent management. Additional limitations include the fact that, for our architecture to be implemented at scale, the participation of federal and regional agencies is required to make up the participating nodes in the blockchain. In addition, data collectors will need to enroll in our platform and allow integration with the framework for the users to be able to give proper consent. Given that the platform will increase transparency and compliance with regulations for data collectors and owners alike, the stakeholders will benefit from engaging with such a platform, ensuring their participation. This means that the success of this platform depends upon the collaboration of several governments and industry partners interested in improving the current security and privacy issues.

Another limitation of our work is that the current version of the framework is not prepared to share health care data between data custodians and consumers. The platform serves as a tool for obtaining, managing, and consulting informed consent and SDM information. Custodians can use the platform to ensure that a data consumer has collected proper informed consent from the owners of the health care data they are requesting. For future work, we intend to extend our conceptual framework to be capable of offering data brokerage between custodians and consumers.

### Conclusions

AAL technologies have the potential to completely revolutionize how older adults age, minimize risks, and increase independence [17]. However, this must be considered alongside the privacy implications of monitoring technologies [17,18]. Unfortunately, it is currently challenging for individuals to successfully manage their consent for data collection [1]. Blockchain is a novel technology that provides immutability and decentralization, allowing increased transparency across processes [1,32]. In this work, we modeled the trust issues existing in the consent management process of AAL technologies and proposed a conceptual framework based on blockchain to mitigate the identified trust issues. The proposed framework can be applied in different domains that deal with sensor data, such as drug supply chain [78-80] and environmental surveillance [1]. The instantiation of the platform is still in the early stages of development, but the first implementation of a fully functional application prototype has already been developed. The prototype allows researchers to request informed consent from AAL patients, and each new informed consent is wrapped into a transaction and sent into the HFN. Organizations validate and endorse the new transaction by using chaincode before it is added to the ledger. After the new block is added, participants of the network can query the ledger to check for informed consent validity before sharing patients’ AAL data. Finally, although our prototype is currently not open source, we hope that researchers will use our framework to create their own blockchain applications.

## Abbreviations

AAL: active assisted living
C1: contract one
C2: contract two
DLT: distributed ledger technology
EHR: electronic health record
FDA: Food and Drug Administration
GDPR: General Data Protection Regulation
HF: hyperledger fabric
HFN: hyperledger fabric network
HIPAA: Health Insurance Portability and Accountability Act
IoT: internet of things
MSP: membership service provider
PIPEDA: Personal Information Protection and Electronic Documents Act
RESTful: representation state transfer
RPM: remote patient monitoring
SDK: software development kit
SDM: substitute decision maker

## References

[1] Bublitz FM, Oetomo A, Sahu KS, Kuang A, Fadrique LX, Velmovitsky PE, Nobrega RM, Morita PP (2019). Disruptive technologies for environment and health research: an overview of artificial intelligence, blockchain, and internet of things. Int J Environ Res Public Health.

[2] Piwek L, Ellis DA, Andrews S, Joinson A (2016). The rise of consumer health wearables: promises and barriers. PLoS Med.

[3] Wei J (2014). How wearables intersect with the cloud and the internet of things : considerations for the developers of wearables. IEEE Consumer Electron Mag.

[4] Mohun J (1989). Design for health. Nurs Times.

[5] (2016). Life in the Fast Lane: How are Canadians managing?. Satistics Canada.

[6] Medeiros H (2016). [ 57% of the Brazilian population uses smartphones, says study]. Exame.

[7] (2020). Smartphone penetration in Argentina. Statista.

[8] Silver L, Smith A, Jonhson C, Jiang J, Anderson M, Rainie L (2019). Mobile Connectivity in Emerging Economies. Pew Research Center.

[9] (2018). Global smartwatch unit sales 2014-2018. Statista.

[10] (2020). Wearables - Canada. Statista.

[11] de Arriba-Pérez F, Caeiro-Rodríguez M, Santos-Gago J (2016). Collection and processing of data from wrist wearable devices in heterogeneous and multiple-user scenarios. Sensors (Basel).

[12] Hovsepian K, al'Absi M, Ertin E, Kamarck T, Nakajima M, Kumar S (2015). cStress: towards a gold standard for continuous stress assessment in the mobile environment. Proc ACM Int Conf Ubiquitous Comput.

[13] (2016). ecobee Launches ‘Donate Your Data,’ Connects Customers to Leading Energy Researchers to Address Climate Change. Ecobee.

[14] (2020). Ecobee.

[15] Barrett MA, Humblet O, Hiatt RA, Adler NE (2013). Big data and disease prevention: from quantified self to quantified communities. Big Data.

[16] Vijayalakshmi A, Jose DV (2020). Internet of Things for Ambient-Assisted Living—An Overview. Internet of Things Use Cases for the Healthcare Industry.

[17] Fadrique LX, Rahman D, Morita PP (2018). The Active Assisted Living Landscape in Canada. CSA Group.

[18] Novitzky P, Smeaton AF, Chen C, Irving K, Jacquemard T, O’Brolcháin F, O’Mathúna D, Gordijn B (2014). A review of contemporary work on the ethics of ambient assisted living technologies for people with dementia. Sci Eng Ethics.

[19] (2014). Government of Canada — Action for Seniors report. Government of Canada.

[20] Faiola A, Papautsky EL, Isola M (2019). Empowering the aging with mobile health: a mHealth framework for supporting sustainable healthy lifestyle behavior. Curr Probl Cardiol.

[21] (2017). Important Facts about Falls | Home and Recreational Safety. CDC Injury Center.

[22] Barney R, Antisdel M. (2013). Common Problems in Informed Consent. Yale University.

[23] O’Connor Y, Rowan W, Lynch L, Heavin C (2017). Privacy by design: informed consent and internet of things for smart health. Procedia Comput Sci.

[24] Gupta U (2013). Informed consent in clinical research: revisiting few concepts and areas. Perspect Clin Res.

[25] Ekblaw MC (2017). Medrec: Blockchain for Medical Data Access, Permission Management and Trend Analysis Signature redacted Signature. Massachusetts Institute of Technology.

[26] Benchoufi M, Ravaud P (2017). Blockchain technology for improving clinical research quality. Trials.

[27] Knifed E, Lipsman N, Mason W, Bernstein M (2008). Patients' perception of the informed consent process for neurooncology clinical trials. Neuro Oncol.

[28] Williams E (2014). Informed consent in evaluation: informed of what, exactly?. LCJ.

[29] Heyland DK, Cook DJ, Rocker GM, Dodek PM, Kutsogiannis DJ, Peters S, Tranmer JE, O'Callaghan CJ (2003). Decision-making in the ICU: perspectives of the substitute decision-maker. Intensive Care Med.

[30] Mainetti L, Patrono A, Secco A, Sergi I (2016). An IoT-aware AAL system for elderly people.

[31] Almeida A, Mulero R, Rametta P, Urošević V, Andrić M, Patrono L (2019). A critical analysis of an IoT—aware AAL system for elderly monitoring. Future Gener Comput Syst.

[32] Pineda D, Urban MC (2018). Inside the Black Blocks. Mowat Centre.

[33] Zheng X, Mukkamala RR, Vatrapu R, Ordieres-Mere J (2018). Blockchain-based personal health data sharing system using cloud storage. IEEE.

[34] Guegan D (2017). Public Blockchain versus Private blockhain. HAL archives.

[35] Wright CS (2008). Bitcoin: a peer-to-peer electronic cash system. SSRN J.

[36] Agbo C, Mahmoud Q, Eklund J (2019). Blockchain technology in healthcare: a systematic review. Healthcare (Basel).

[37] Zheng Z, Xie S, Dai H, Chen X, Wang H (2017). An Overview of Blockchain Technology: Architecture, Consensus, and Future Trends.

[38] Androulaki E, Barger A, Bortnikov V, Cachin C, Christidis K, de Caro A, Enyeart D, Ferris C, Laventman G, Manevich Y, Muralidharan S, Murthy C, Nguyen B, Sethi M, Singh G, Smith K, Sorniotti A, Stathakopoulou C, Vukolić M, Cocco S.W., Yellick J (2018). Hyperledger fabric: a distributed operating system for permissioned blockchains. Proceedings of the Thirteenth EuroSys Conference.

[39] Bahga A, Madisetti VK (2016). Blockchain platform for industrial internet of things. J Softw Eng Appl.

[40] Underwood S (2016). Blockchain beyond bitcoin. Commun ACM.

[41] Nakamoto S (2008). Bitcoin: A Peer-to-Peer Electronic Cash System. Bitcoin.

[42] Wang H, Wang Y, Cao Z, Li Z, Xiong G (2019). An overview of blockchain security analysis. Cyber Security.

[43] Gorenflo C, Golab L, Keshav S (2019). Mitigating trust issues in electric vehicle charging using a blockchain. Proceedings of the Tenth ACM International Conference on Future Energy Systems.

[44] IEC - SyC AAL: Active Assisted Living. International Electrotechnical Comission.

[45] Morgan-Linnell SK, Stewart DJ, Kurzrock R (2014). US Food and Drug Administration inspections of clinical investigators: overview of results from 1977 to 2009. Clin Cancer Res.

[46] Statement by Marisol Touraine-Press conference-Clinical trial accident-progress report. Ministry of Solidarity and Health.

[47] (2018). Blockchain and GDPR: How blockchain could address five areas associated with GDPR compliance. IBM.

[48] Hyperledger Fabric. Hyperledger.

[49] Introduction. Hyperledger.

[50] Hyperledger Fabric Network. Hyperledger.

[51] Glossary. Hyperledger.

[52] Peers. Hyperledger.

[53] The Ordering Service. Hyperledger.

[54] Membership Service Provider (MSP). Hyperledger.

[55] Ledger. Hyperledger.

[56] Chaincode for Developers. Hyperledger.

[57] Ethereum Whitepaper. Ethereum.

[58] Bocek T, Rodrigues BB, Strasser T, Stiller B (2017). Blockchains everywhere - A use-case of blockchains in the pharma supply-chain. Proceedings of the IM 2017 - 2017 IFIP.

[59] IBM Blockchain Platform. IBM.

[60] Greenspan G (2015). MultiChain Private Blockchain-White Paper. MultiChain.

[61] HydraChain. GitHub.

[62] Mcconaghy T, Marques R, Muller A, De Jonghe D, McConaghy T, McMullen G, Henderson R, Bellemare S, Granzotto A (2016). BigchainDB: A Scalable Blockchain Database - White Paper. MyCourses.

[63] Blockchain network. Hyperledger.

[64] Morita PP, Sethumadhavan A, Sasangohar F (2020). Chapter 5 - Design of mobile health technology. Design for Health.

[65] Goyal S, Morita P, Lewis GF, Yu C, Seto E, Cafazzo JA (2016). The systematic design of a behavioural mobile health application for the self-management of type 2 diabetes. Can J Diabetes.

[66] Morita PP, Yeung MS, Ferrone M, Taite AK, Madeley C, Stevens Lavigne A, To T, Lougheed MD, Gupta S, Day AG, Cafazzo JA, Licskai C (2019). A patient-centered mobile health system that supports asthma self-management (breathe): design, development, and utilization. JMIR Mhealth Uhealth.

[67] Sultan M, Kuluski K, McIsaac WJ, Cafazzo JA, Seto E (2019). Turning challenges into design principles: telemonitoring systems for patients with multiple chronic conditions. Health Informatics J.

[68] Omran Y, Henke M, Heines R, Hofmann E (2017). Blockchain-driven supply chain finance: towards a conceptual framework from a buyer perspective. International Purchasing and Supply Education and Research Association 2017.

[69] Carney D, Çetintemel U, Cherniack M, Convey C, Lee S, Seidman G, Stonebraker M, Tatbul N, Zdonik S (2002). Monitoring streams: a new class of data management applications. Proceedings of the 28th international conference on Very Large Data Bases.

[70] Fingberg J, Hansen M, Hansen M, Krasemann H, Iacono LL, Probst T, Wright J (2006). Integrating data custodians in ehealth grids - A digest of security and privacy aspects Digest of Security and Privacy Aspects. Informatik 2006.

[71] Xia Q, Sifah EB, Asamoah KO, Gao J, Du X, Guizani M (2017). MeDShare: trust-less medical data sharing among cloud service providers via blockchain. IEEE Access.

[72] Ongaro D, Ousterhout J (2014). In search of an understandable consensus algorithm. Proceedings of USENIX ATC ’14.

[73] (2018). Fabric SDK for node.js. Hyperledger.

[74] (2018). Substitute Decision Makers and Naming a Power of Attorney for Personal Care. University Health Network.

[75] Takahashi D Hu-manity.co uses IBM blockchain to give you the right to control your personal data. VentureBeat.

[76] Alexandre A New Bitfury Joint Project to Manage Medical Data Permissions With Blockchain Tech. Cointelegraph.

[77] Bitfury announces blockchain-based consent management system; partners with Hancom to distribute Crystal platform. Tokenpost.

[78] Zanghi E, Do Coutto Filho MB, Stacchini de Souza JC (2019). Conceptual framework for blockchain-based metering systems. Multiagent Grit Syst.

[79] (2018). Building blockchains for a better planet. PwC.

[80] Siwicki B The next big thing in pharmacy supply chain: Blockchain. Healthcare IT News.

